# Dynamic High-Sensitivity Quantitation of Procollagen-I by Endogenous CRISPR-Cas9 NanoLuciferase Tagging

**DOI:** 10.3390/cells9092070

**Published:** 2020-09-10

**Authors:** Ben C. Calverley, Karl E. Kadler, Adam Pickard

**Affiliations:** 1 Wellcome Centre for Cell-Matrix Research, Faculty of Biology, Medicine & Health, University of Manchester, Manchester Academic Health Science Centre, Manchester M13 9PT, UK; ben.calverley@postgrad.manchester.ac.uk; 2 School of Mathematics, Faculty of Science and Engineering, School of Mathematics, University of Manchester, Manchester M13 9PT, UK

**Keywords:** circadian clock, hydroxyproline, rhythm, collagen, GFP, bioluminescence, quantitative biolog

## Abstract

The ability to quantitate a protein of interest temporally and spatially at subcellular resolution in living cells would generate new opportunities for research and drug discovery, but remains a major technical challenge. Here, we describe dynamic, high-sensitivity protein quantitation technique using NanoLuciferase (NLuc) tagging, which is effective across microscopy and multiwell platforms. Using collagen as a test protein, the CRISPR-Cas9-mediated introduction of nluc (encoding NLuc) into the Col1a2 locus enabled the simplification and miniaturisation of procollagen-I (PC-I) quantitation. Collagen was chosen because of the clinical interest in its dysregulation in cardiovascular and musculoskeletal disorders, and in fibrosis, which is a confounding factor in 45% of deaths, including those brought about by cancer. Collagen is also the cargo protein of choice for studying protein secretion because of its unusual shape and size. However, the use of overexpression promoters (which drowns out endogenous regulatory mechanisms) is often needed to achieve good signal/noise ratios in fluorescence microscopy of tagged collagen. We show that endogenous knock-in of NLuc, combined with its high brightness, negates the need to use exogenous promoters, preserves the circadian regulation of collagen synthesis and the responsiveness to TGF-β, and enables time-lapse microscopy of intracellular transport compartments containing procollagen cargo. In conclusion, we demonstrate the utility of CRISPR-Cas9-mediated endogenous NLuc tagging to robustly quantitate extracellular, intracellular, and subcellular protein levels and localisation.

## 1. Introduction

Quantitation of DNA and RNA is routine in research and diagnostic laboratories, and makes use of base pair hybridisation to ensure specificity and identification. Similar approaches are not available for proteins. Methods such as ELISA immunoassays and western blotting are widely used to estimate levels of proteins, but spatial resolution is lost, and they are unsuitable for live cell studies where dynamic readouts are required. In this regard, the use of fluorescent proteins and chemical tags has revolutionised cell biology, but quantitation through fluorescence is not without technical difficulties associated with quenching, extensive wash-out, and the influence of the local environment on the fluorescence signal. Low fluorescence signals can be overcome with the use of strong exogenous promoters, but these disrupt the endogenous behaviour of the protein under study.

Bioluminescence produced when luciferase hydrolyses luciferin-based substrates offers a practical alternative to using fluorescent tags. When tagged to a protein of interest, luciferase emits visible light in the presence of a suitable substrate. Hall and coworkers used a small luciferase subunit from the deep-sea shrimp *Oplophorus gracilirostris* to produce NanoLuciferase (NLuc), which produces more photons than either firefly or Renilla luciferases when used in combination with a novel imidazopyrazinone substrate, furimazine [1]. In our study we used CRISPR-Cas9 to fuse NLuc to the N-terminus of procollagen-I (PC-I), which is the precursor of collagen-I and the most abundant protein in vertebrates [2]. Collagen-I is a triple helical protein [3] that occurs in the extracellular matrix as elongated fibrils that are established during development [4] and remain throughout adulthood without turnover [5] in the presence of a sacrificial pool of collagen that is under circadian control [6]. Although the scaffolding function of collagen I is essential for tissue integrity, excess collagen causes tissue damage in fibrosis (scarring) and is associated with aggressive cancers [7,8] and 45% of deaths [9]. Thus, collagen is of broad clinical importance, from regenerative medicine, in which elevating collagen synthesis is needed to build tissue, to fibrosis, in which inhibiting collagen synthesis is required to stop loss of tissue function. However, the identification of drugs to either increase or decrease collagen levels is hampered by the lack of suitable technologies for measuring collagen levels in cell culture. Collagen-I contains ~13.6% hydroxyproline [10], and assay of hydroxyproline has become the gold standard for quantifying tissue collagen. However, hydroxyproline also occurs in the 27 other collagens [11], noncollagenous triple helical proteins (reviewed by [12]), and elastin [13], which is difficult to take into account when using hydroxyproline to estimate levels of collagen-I. Moreover, the assay is destructive and unsuitable for time-resolved studies of collagen synthesis in single cells. Proteomics [6], western blotting, and the use of fluorescent tags (e.g. green fluorescence protein, GFP) are either destructive or require the use of overexpression promoters to provide good signal/noise ratios. Furthermore, these approaches cannot quantify the rapid synthesis and secretion of collagen, for which pulse-chase approaches (using ^3^H- and ^14^C-biosynthetic labelling) have shown to occur within minutes [14]. In our study, we show that the light produced by NLuc is sufficiently bright to obtain dynamic quantitative information on the number of endogenous collagen-I molecules trafficking through living cells and being secreted and incorporated into the extracellular matrix.

## 2. Materials and Methods

### 2.1. Cell Culture

NIH3T3 mouse embryonic fibroblasts and subsequently CRISPR edited cells were maintained in DMEM (Dulbecco’s Modified Eagle Medium) supplemented with heat-inactivated 10% new-born calf serum, 1% l-glutamine, and 1% penicillin and streptomycin. The cells were kept at 37 °C in humidified incubators with 5% CO_2_. They were passaged using trypsin. For 96-well plate reader recordings, cells were seeded into a white plastic plate, in the cell culture medium described above. Furimazine substrate was then added as required, at levels of 0.25 μL per 100 μL medium unless otherwise specified.

### 2.2. Generation of Split GFP Expressing Stable Cells

To detect CRISPR edited cells, we included a split GFP tag developed in the Bo Huang lab [15]. The sfGFP1-10 barrel was synthesised and cloned into a lentiviral vector (Vectorbuilder Inc., Chicago, IL, USA), and further subcloned into a CMV driven vector (pLenti CMV V5-LUC Blast (w567-1) was a gift from Eric Campeau (Addgene (Watertown, MA, USA) plasmid #21474; http://n2t.net/addgene:21474; RRID:Addgene_21474 [16])). Briefly, FLuc was removed from the vector by digesting with BstXI. sfGFP1-10 was PCR amplified with the addition of a signal peptide to target expression to the endoplasmic reticulum (ER), using the primers listed in Table S1, and assembled using a Gibson Assembly master mix (New England Biolabs (NEB), Ipswich, MA, USA). Then, 5 µg pLV-ERsfGfp1-10 was transfected into 293T cells, along with 2.5 µg VSVG, 2.5 µg pRSV-Rev, and 2.5 µg pMDLg/pRRE, using a 3:1 ratio of PEI:DNA, to generate lentivirus. Next, the medium was collected 24–48 h posttransfection, filtered through a 0.45 µm filter, and added to NIH3T3 cells with 8 µg/mL polybrene. After overnight infection, fresh medium was added for 8 h before selecting for 72 h in 2.5 µg/mL Blasticidin to generate NIH3T3-ERsfGFP1-10.

### 2.3. CRISPR Editing

The NanoLuciferase sequence was taken from the pNL1.1 vector map (Promega Corporation, Madison, WI, USA); this and the sfGFP11 sequence [15] were synthesised as a gBLOCK from Integrated DNA Technologies (IDT, Coralville, USA) (Table S2). The 5′ and 3′ homology arms were generated by PCR amplification using a repair template previously used to introduce a Dendra2 tag into the *Col1a2* locus [17] using primers in Table S1. The NanoLuciferase gBLOCK and homology arms were joined using Gibson assembly master mix (NEB) and transformed into Stbl3 bacteria, resulting in the generation of an sfGFP11-NLuc *Col1a2* repair template (Table S2).

**Figure 1:**
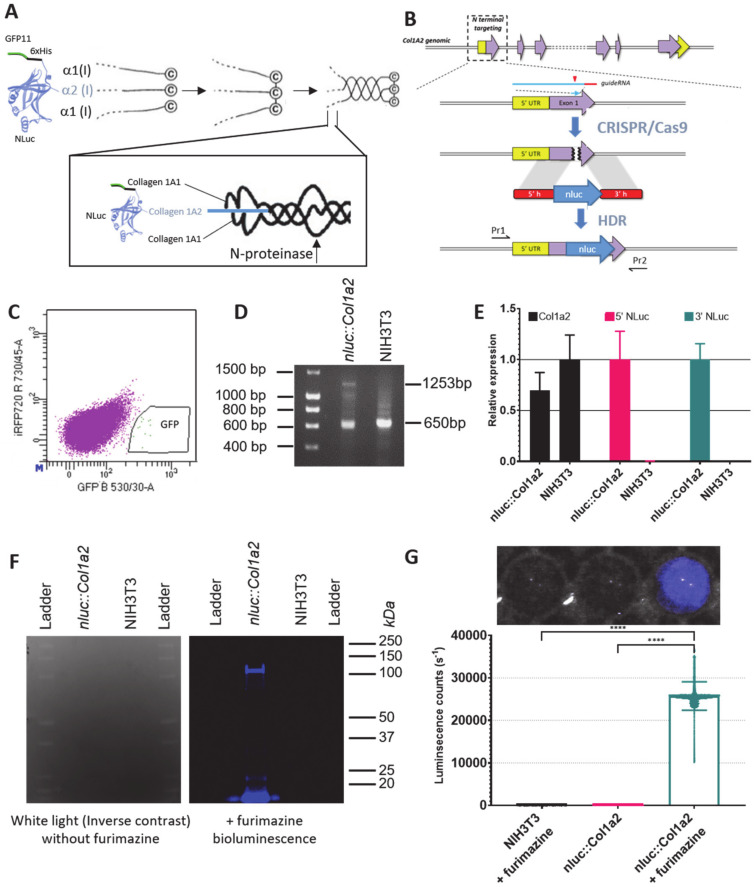
Quantitative CRISPR/Cas9 editing strategy. (**A**,**B**) Tagging strategy to allow quantitation of PC-I in NIH3T3 fibroblasts with the introduction of a multifunctional tag at the N-terminus of proα2(I) to allow detection of edited cells, and the small luciferase, NLuc. Schematic not drawn to scale. The estimated molecular weights of each domain are: GFP11 (residues 215-230; 1773 Da), 6xHis (840 Da), NLuc (19095 Da). (**C**) After introduction of the multifunctional tag in exon 1 of *Col1a2* using CRISPR/Cas9, edited cells were sorted based on GFP fluorescence. (**D**) PCR validation of edited DNA in cells isolated from (**C**) using primers in Table S1. (**E**) Real-time PCR of total *Col1a2* transcripts (Black) and edited transcripts in NIH3T3 and *nluc::Col1a2* cells. Primers at the 5′ and 3′ ends of the introduced *nluc* confirmed insertion into the *Col1a2* transcript. Bars show mean ± SD, *n* = three independent experiments. (**F**) In-gel detection of NLuc tagged proα2(I) chain under reducing conditions identified NLuc activity at approximately 140 kDa. (**G**) Imaging and quantitation of the light produced by *nluc::Col1a2* and unedited NIH3T3 cells incubated with the NLuc substrate, Furimazine (Nano-Glo). On a 96-well plate, single wells were imaged using a single-lens reflex camera and quantitation of photon counts was performed using a multiwell plate luminometer. Bar shows means ± SD from *n* = 30 replicate measurements. **** represents *p* = 0.0001, paired Student’s t-Test.

NIH3T3-ERsfGFP1-10 were used to perform CRISPR editing. First, 1 µg of repair template was transfected into 200,000 cells using a 3:1 ratio Fugene6:DNA (Promega Corporation, Madison, WI, USA). After overnight transfection, cells were grown in fresh medium for 6 h, and then transfected with a *Col1a2* crRNA (ACTTACATTGGCATGTTGCT AGG), tracrRNA and Cas9 (IDT), as previously described [17]. crRNA sequences were selected using the Wellcome Trust Sanger Institute Genome Editing database [18]. After overnight transection, cells were grown for 72 h in fresh medium. Cells were sorted based on GFP positivity, and expanded before validating the CRISPR knock-in. Following initial validation of Nluc activity within this population, individual cells were sorted into 96-well plates, using forward and side scatter to identify individual cells. Single cell clones were maintained and expanded as described above. Clone #2 was used for all figures with the exception of Figure 1 and Figures S1–S3, S6, and S7.

### 2.4. DNA and RNA Validation of CRISPR Editing 

Knock-in of NanoLuciferase was validated initially using a Nano-Glo assay, and then validated at the DNA level by PCR across the gRNA cut site using primers ValF and ValR (Table S1). Edited cells were trypsinised, pelleted, and lysed using the Hotshot DNA isolation method. To further ensure the knock-in, RNA was isolated from knock-in cells and quantitative PCR was performed from the unedited 3′ end of the Col1a2 transcript into the NanoLuciferase sequence. PCR products were sequenced using Sanger sequencing. Similarly, primers to the 5′ end of the NanoLuciferase sequence and the unedited *Col1a2* region were used to ensure that the reading frame between NanoLuciferase and col1a2 was maintained. 

### 2.5. In-Gel Detection of NLuc Activity

As a further validation of the *nluc::Col1a2* cell line, the molecular weight at which NLuc activity could be detected was determined by 1D gel electrophoresis and in-gel detection of NLuc. *nLuc: Col1a2* cells were trypsinised, pelleted at 1000× *g* for 5 min, and lysed in 8 M urea and 50 mM Tris pH7.5 supplemented with PMSF and phosphatase inhibitors (Sigma). After centrifugation at 12,000× *g* for 5 min, 50 µg protein was loaded onto a 6% Tris-Glycine gel. Proteins were renatured and assayed according to the Nano-Glo^®^ In-Gel Detection System protocol (Promega). Light produced by NLuc was captured using a Chemidoc MP Imager (Bio-Rad Laboratories Ltd., Hercules, CA, USA).

### 2.6. Proteomic Validation of CRISPR Editing

For validation of NLuc integration into the Col1a2 locus, 1L of culture medium from nluc: Col1a2 cells was collected over the course of 2 weeks; cells were grown as described above. Aliquots were frozen at −80 °C until use. The conditioned medium was flowed through a 5 mL His-Trap fast-flow (GE Life Sciences, Marlborough, MA, USA) column at a flow rate of 4 mL/min using an NGC chromatography system (Bio-Rad) with a dedicated sample pump. The column was equilibrated in 20 mM Tris-HCl pH 7.4 with 0.15M NaCl (buffer A). The column was washed by mixing 4% buffer B, which was 20 mM Tris-HCl, 0.15 M NaCl, and 500 mM imidazole (Ultrapure, Thermo Fisher Scientific, Waltham, MA, USA). Bound proteins were eluted with a step gradient of 4% to 100% buffer B in reverse flow at a flow rate of 2 mL/min. Eluted proteins were collected in 0.5 mL fractions. Twenty microliters of each fraction were mixed with the LDS sample loading buffer (Life Technologies, Thermo Fisher Scientific) without reducing agents, heated at 95 °C for 5 min, and then run on a 6% tris-glycine gel. Following Coomassie Blue staining, bands of interest were excised from the gel and dehydrated using acetonitrile followed by vacuum centrifugation. Dried gel pieces were reduced with 10 mM dithiothreitol and alkylated with 55 mM iodoacetamide. Gel pieces were then washed alternately with 25 mM ammonium bicarbonate followed by acetonitrile. This was repeated, and the gel pieces were dried by vacuum centrifugation. Samples were digested with trypsin overnight at 37 °C. Digested samples were analysed by LC-MS/MS using an UltiMate^®^ 3000 Rapid Separation LC (RSLC, Dionex Corporation, Sunnyvale, CA, USA) coupled to an QExactive HF (Thermo Fisher Scientific) mass spectrometer. Peptide mixtures were separated using a gradient from 92% A (0.1% FA in water) and 8% B (0.1% FA in acetonitrile) to 33% B, in 44 min at 300 nL/min, using a 75 mm × 250 μm i.d. 1.7 µM BEH C18, analytical column (Waters Corporation, Milford, MA, USA). Peptides were selected for fragmentation automatically by data dependent analysis. Mass spectrometry was searched using Mascot (Matrix Science Ltd, London, UK), against the Swissprot and Trembl databases with taxonomy of Mouse selected, as well as a custom database including the sequence of NLuc-tagged Col1a2. Data were validated using Scaffold (Proteome Software, Portland, OR, USA).

### 2.7. Quantitation of Absolute Collagen Levels

**Figure 2:**
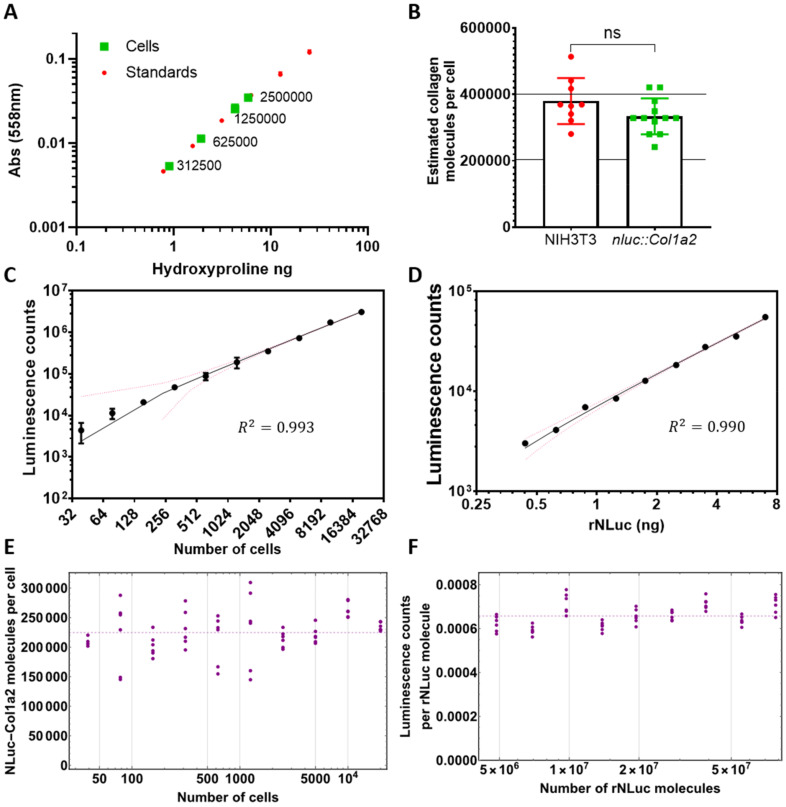
Quantitation of intracellular collagen molecules. (**A**) The quantity of hydroxyproline in known numbers of cells was calculated using known concentrations of hydroxyproline. Greater than 300,000 cells were required to ensure accurate comparison to hydroxyproline standards. (**B**) Estimated numbers of collagen molecules per cell based on hydroxyproline. *n* = 3 independent experiments each conducted in triplicate, NIH3T3 and *n* = 4 independent experiments each conducted in triplicate. Bars show mean ± SD. (**C**) Bioluminescence counts per second of *nluc::Col1a2* cells scales with cell number. *n* = 3 independent assays, each recorded *n* = 4 times (**D**) Correlation of known quantities of recombinant NLuc (rNLuc) with luminescence counts. *n* = 3 independent assays, each recorded *n* = 4 times. (**E**) Across the range of rNLuc concentrations tested, reaction conditions allowed consistent counts per rNLuc molecule to be measured. (**F**) By comparing the bioluminescence from C and D, the number of NLuc-PC-I molecules per cell could be quantified.

Luminescence activity was recorded from known masses of rNLuc protein in culture medium when treated with Furimazine. The same procedure was carried out for *nluc::Col1a2* cells at differing confluence levels. The results can be seen in Figure 2A. The rNLuc protein has a mass of 54.254 kDa; therefore, 1 μg contains 1.11 × 10^13^ NLuc molecules, and we can convert from mass to concentration. We then used linear regression to generate equations for the relationship between total rNLuc molecule counts and luminescence counts, and between number of cells and luminescence counts (Figure 2C–F). This procedure was repeated for cells in bioluminescence imaging.

### 2.8. Hydroxyproline Assay

The protocol for the hydroxyproline assay to quantify collagen amounts and correlated to luminescence was as follows. *nluc::Col1a2* cells were trypsinised, washed with PBS, counted, and pelleted. NLuc-PC-I activity was assessed in a serial dilution of the cell pellets. Matching numbers of cells were pelleted and frozen at −20 °C for hydroxyproline quantitation. Hydroxyproline was measured using methods previously described [19]. Briefly, 100 µL 6M HCl was added to the cell pellet and incubated at 100 °C overnight. Samples were cooled to room temperature and spun at 12,000× *g* for 3 min to remove residual charcoal. Each sample (50 μL) was mixed with chloramine T (450 μL) and incubated at room temperature for 25 min. Ehrlich’s reagent (500 μL) was added to each sample and incubated at 65 °C for 10 min. All samples were compared to hydroxyproline standards treated identically. The absorbance of 100 μL was measured a 96-well plate and absorbance at 558 nm read on a H1 plate reader (BioTek, Winooski, VT, USA).

### 2.9. Bioluminescence Imaging

Bioluminescence imaging of recombinant NLuc and *nluc::Col1a2* cells was performed in black walled µ-Plate 96-well plates (iBidi, Munich, Germany). All imaging was performed in DMEM containing 10% FBS. For imaging rNLuc, wells containing NIH3T3 cells were used to ensure that the focal point was in the same position as when imaging *nluc::Col1a2* cells. Imaging was performed at 37 °C using a 40× oil objective on a Zeiss LSM880 microscope (Zeiss, Oberkochen, Germany) fitted with a Hamamatsu ImageEM electron multiplying CCD (Hamamatsu, Hamamatsu, Japan). One-minute integration times were used for all samples 

## 3. Results

### 3.1. CRISPR-Cas9 Editing of Col1a2

Mouse NIH3T3 is a mouse embryonic fibroblast that has been immortalised by a standardised passaging procedure [20]. These fibroblasts produce both proα1(I) and proα2(I), which make up the heterotrimeric type I procollagen molecule, and also assemble the type I collagen into fibrils, making them suitable for studying the entire biosynthesis and fibril assembly processes. In order to monitor how cells produce type I collagen, we introduced the DNA sequence encoding NLuc into the *Col1a2* locus. In order to identify and clonally select for *nluc::Col1a2* edited fibroblasts, we introduced a multifunctional tag (Figure 1A). This tag was inserted where the ER-targeting signal recognition sequence (SP) of *Col1a2* is retained, and encodes a split GFP (GFP11) sequence, 6 histidine (6xHis) residues for PC-I capture, and NLuc. Selection of edited cells was performed using fibroblasts expressing the GFP barrel, GFP1-10, which generates a fluorescent protein when bound by GFP11 (Figure 1C). These GFP-positive cells were expanded and assessed for further evidence of CRISPR editing before single cell clones were generated. Edited cells did not have any obvious defects in proliferation (Figure S1B–C). CRISPR editing in the GFP-positive population was confirmed by PCR from genomic DNA (Figure 1D) and quantitation and sequencing of RNA transcripts across the junctions of *nluc* and *Col1a2* (Figure S1A). Sequencing of the reverse transcribed-PCR products confirmed the introduction of *nluc* in-frame with *Col1a2* (Figure S2D). Secretion of NLuc-PC-I was confirmed by His-trap capture of the protein from the medium of edited cells (Figure S2A). A peptide spanning the junction of NLuc and proα2(I) was identified by LC-MS/MS (Figure S2D). Incorporation of NLuc into the heterotrimer of PC-I was also confirmed in high molecular weight complexes, where association with proα1(I) was identified (Figure S2B). Under reduced conditions, NLuc-PC-I was identified by in-gel detection of NLuc activity at approximately 140 kDa (Figure 1F). The culture medium from *nluc::Col1a2* fibroblasts was passed over a Ni^2+^ chelating column and bound proteins were eluted with imidazole. The fractions were separated by SDS-PAGE, the gel was stained with Coomassie blue, and protein bands were subjected to LC-MS/MS for protein identification (Figure S2C). The results showed the presence of intact NLuc-PC-I, NLuc-pCcollagen-I (where the C-propeptide has been removed) and mature collagen-I. The presence of free NLuc showed that procollagen N-proteinases were capable of cleaving NLuc-PC-I, which is a good indicator that NLuc-PC-I secreted by *nluc::Col1a2* fibroblasts was triple helical [21].

To demonstrate the ease of detecting NLuc-PC-I, an SLR camera was used to capture the light produced by a single well of a 96-well plate containing *nluc::Col1a2* cells, following the addition of Furimazine (Figure 1G). As endogenous NLuc activity generated a remarkably bright signal, we were required to optimise the plasticware for the assay. Black, white, and clear walled 96-well plates were tested to evaluate the spillover between wells. White walled plates reduced spillover of the produced light between wells and gave the maximal signal. White walled plates were therefore used in all subsequent 96-well plate reader experiments (Figure S3).

### 3.2. Quantitation of NLuc-PC-I

As a first experiment, we used the chloramine-T colorimetric method to quantify the amount of hydroxyproline synthesised by single cell clones of *nluc::Col1a2* fibroblasts (Figure S4). In our hands, at least 300,000 cells were required to synthesise sufficient collagen to be detected using this method (Figure 2A). Next, we compared known numbers of *nluc::Col1a2* cells and known numbers of matched 3T3 cells, and measured hydroxyproline in the cell layer from each set. The results showed that CRISPR-Cas9 editing of the cells did not alter the ability of the cells to synthesise collagen (Figure 2B). We cultured *nluc::Col1a2* cells, added Furimazine, and measured the resultant luminescence (Figure 2C). These experiments demonstrated the high sensitivity of NLuc detection, compared to measurement of hydroxyproline, to detect PC-I synthesis. To be able to quantitate the number of NLuc-PC-I molecules synthesised per cell, we prepared a standard curve of luminescence from recombinant NLuc (rNLuc) in the presence of Furimazine (Figure 2D). An important consideration was whether we could infer a direct correlation between luminescence produced by rNLuc in a well-mixed solution, and NLuc bound to collagen within cells and subcellular compartments. Direct comparison of lysed and unlysed cells indicated that there was no significant difference in the time taken for luminescence levels to peak following the addition of Furimazine (Figure S4). Differences in the absolute level of luminescence were observed; this was explained by differences in the activity of rNLuc in lysis buffer versus DMEM medium. The outcome of these experiments was confidence that we could correlate luminescence levels recorded from known numbers of NLuc molecules to luminescence levels recorded from unknown numbers of NLuc-PC-I molecules in cells or culture medium. By bringing the luminescence and cell number data together, we were able to describe a relationship converting luminescence to numbers of NLuc-PC-I molecules, and showed that luminescence was linear over a range of 39 to 20,000 cells (Figure 2E). Of note, the luminescence counts per rNLuc molecule were constant over a wide range of rNLuc molecules without noticeable quenching or amplification (Figure 2F). Furthermore, a consistent value of 228,000 (median, 3 s.f.) and 225,000 (mean, given by dashed line in Figure 2E, 3 s.f.) was obtained for numbers of NLuc-PC-I molecules per cell after correlation of luminescence from cells and rNLuc across five orders of magnitude.

### 3.3. Direct Imaging and Quantitation of NLuc-PC-I in Cells

**Figure 3:**
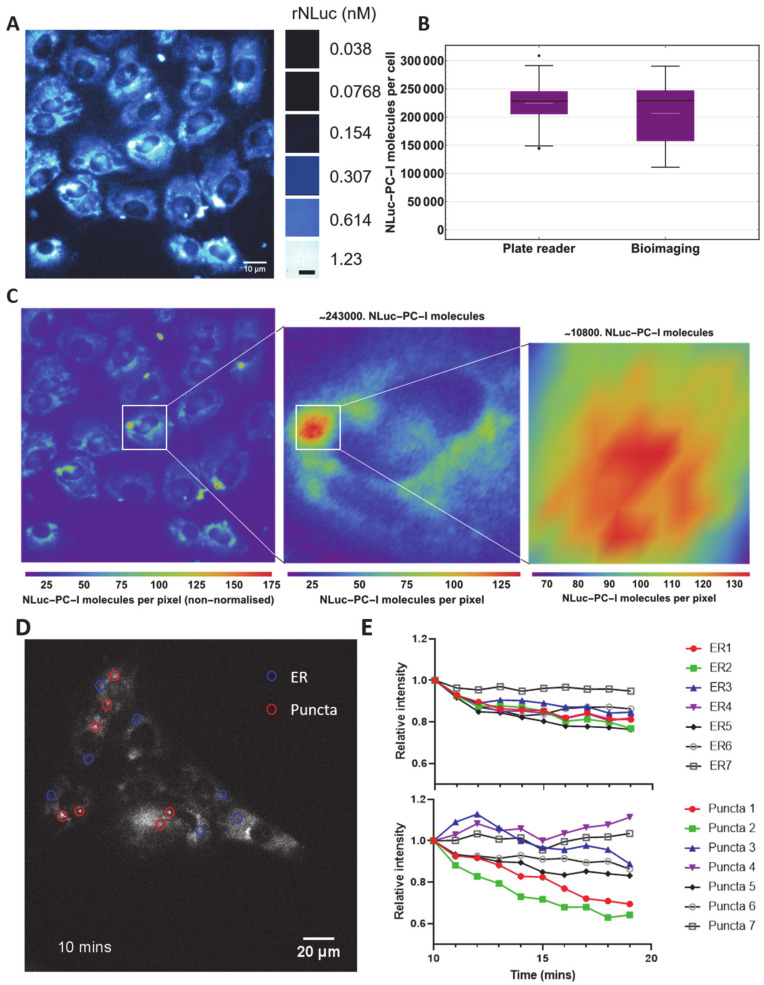
Quantitation at single cell and subcellular levels. (**A**) Bioluminescence imaging of *nluc::Col1a2* cells. Colourised bioluminescence image of NLuc-PC-I immediately following the addition of the substrate Furimazine to 21 cells. Images were taken every minute for 5 min, and the data summed. Adjacent, summed images of rNLuc used for quantitative correlation, scale bar represents 100 µm. Analysis of images is shown in Figure S4. (**B**) Quantitation of NLuc-PC-I molecules per cell using bioluminescence imaging of 21 cells were compared to estimates from all plate reader measurements. The black line within the box shows the median value, and the white dash the mean value. The fences show maximum and minimum values (excluding outliers). (**C**) Scaled image of (**A**) showing the number of NLuc-PC-I molecules per pixel. NLuc-PC-I was found to be concentrated in puncta, quantitation of a single subcellular vesicle containing 10,800 molecules at a concentration of 0.231 mg/mL or 0.479 μM, assuming a spherical vesicle. (**D**) Bioluminescence imaging of nluc::Col1a2 cells with regions of endoplasmic reticulum (ER, blue circles) and puncta indicated (red circles). (**E**) The change in bioluminescence over 10 min of imaging in the seven indicated regions of ER and puncta is shown in (**D**).

Next, we wanted to know if we could quantitate numbers of NLuc-PC-I molecules in bioluminescence microscopy images of the cells. This would provide quantitative information on PC-I trafficking and allow us to assess the sensitivity of collagen-DyProQ. By correlating bioluminescence from known amounts of rNLuc (Figure S6), we were able to determine the number of NLuc-PC-I molecules in bioluminescence images. The total luminescence in each cell within the field of view could then be individually calculated and converted to the total number of NLuc-PC-I molecules per cell (Figure 3A). The results showed a mean of 207,000 (3 s.f.) and a median of 229,000 (3 s.f.) NLuc-PC-I molecules per cell (Figure 3B). These estimates of NLuc-PC-I molecules per cell from bioimages were in strong agreement with the estimates obtained using a plate reader (differing by less than 10% in mean values, and less than 1% in median values). A Student’s t-test comparing the two datasets showed that the difference in the means was not significant (*p* > 0.5). A range of 111,000 to 290,000 (3 s.f.) NLuc-PC-I molecules per cell was observed, representing a 62% variation in cellular collagen levels. We noticed bright luminescence in subcellular vesicles (see for example highlighted region in Figure 3C). From measurements of photon counts, we were able to estimate ~10,800 NLuc-PC-I molecules in the vesicle shown. If we assume the vesicle to be spherical (diameter 4.15 µm (3 s.f.)), then the concentration of NLuc-PC-I within this vesicle is ~ 0.231 mg/mL (3s.f.). We observed higher variability in the bioimaging single cell quantitation in Figure 3B. This was due to the fact that each data point in the bioimaging chart comes from an individual cell, whereas each data point in the plate reader chart represents a mean count per cell from a well containing 39–20,000 cells.

Intracellular NLuc-PC-I was also imaged over time at high temporal resolution, allowing for dynamic protein quantitation of NLuc-PC-I in moving vesicles (Figure 3D and Supplementary Video S1). It was possible to track the movement of individual vesicles and to estimate their size and the number of NLuc molecules they contained. We recorded time-lapse images of the cells (recording for 20 min at 1-min intervals, Figure 3E) and noticed that the luminescence from some puncta increased for 20 min, whereas light levels coming from other puncta remained constant and others faded. Furthermore, the intensity of light from puncta was greater than that from the endoplasmic reticulum (ER), and the intensity of light emanating from the ER decreased during the time series. Presumably, these results may be explained by NLuc-PC-I exiting ER and being transported to sites within the cell for storage, degradation, or secretion.

### 3.4. Circadian Fluctuations of Procollagen-I

**Figure 4:**
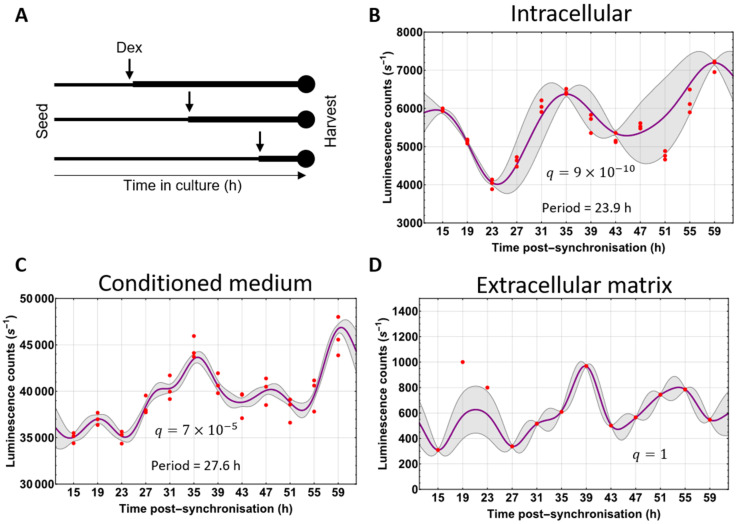
Circadian fluctuations in cellular NLuc-PC-I. (**A**) Schematic of experiments performed to assess circadian fluctuations in PC-I in *nluc::Col1a2* cells whilst maintaining consistent cell numbers. (**B**) The levels of cellular NLuc-PC-I activity over 48 h, MetaCycle analysis indicated a 23.9 h periodic fluctuation in cellular procollagen. Graph shows *n* = 3 independent replicate data points (red), Gaussian process predicted function (purple), and standard deviation of the Gaussian process (grey). (**C**) The levels of secreted NLuc-PC-I activity over 48 h. MetaCycle analysis indicated a 27.8 h periodic fluctuation in secreted NLuc-PC-I. The cellular and secreted NLuc-PC-I levels follow the same pattern. Graph shows *n* = 3 independent replicates. (**D**) The incorporation of NLuc-collagen-I into the extracellular matrix was assessed following decellularisation at each time. Whilst levels fluctuated over 48 h, MetaCycle analysis did not indicate a periodic incorporation of NLuc-collagen-I into the extracellular matrix. Graph shows *n* = 3 independent replicates.

We noted that the levels of NLuc-PC-I luminescence, when monitored by bioimaging, varied more than plate reader measurements, suggesting that each cell has variable amounts of Col1a2 (Figure 3A). It has recently been shown that PC-I levels in tendon fluctuate rhythmically for 24 h under the control of the circadian clock [6]. Therefore, to explore the possibility that the variation in NLuc levels observed in individual cells could reflect differences in PC-I levels in cells at different stages of the circadian cycle, we synchronised *nluc::Col1a2* cells every 4 h (Figure 4A) and measured luminescence as a function of time postsynchronisation. Intracellular NLuc-PC-I exhibited a strong circadian rhythm as shown by MetaCycle [22] (23.9 h) and a low Benjamini-Hochberg [23] q-value (9 × 10^−10^) (Figure 4B). The time of peak levels of intracellular luminescence was 12.2 h postsynchronisation (estimated to be circadian time CT0), which aligns well with observations of peak PC-I levels in tendon in vivo [6]. These findings provided direct evidence that the circadian clock influences the synthesis of NLuc-PC-I in *nluc::Col1a2* cells. Rhythmic fluctuations were also observed for secreted NLuc-PC-I, having a period of 27.6 h (3 s.f.) and a q-value 7 × 10^−5^ (Figure 4C). Here, overall NLuc levels increased relative to the time after synchronisation, presumably because of PC-I accumulation in the culture medium during the recording period. Fluctuations in NLuc in the matrix fraction were not 24 h rhythmic (Figure 4D); this was presumably a result of NLuc-collagen accumulation in the form of fibrils and the transport of cleaved N-propeptides.

### 3.5. NLuc-PC-I Response to Known Collagen Modulators

**Figure 5:**
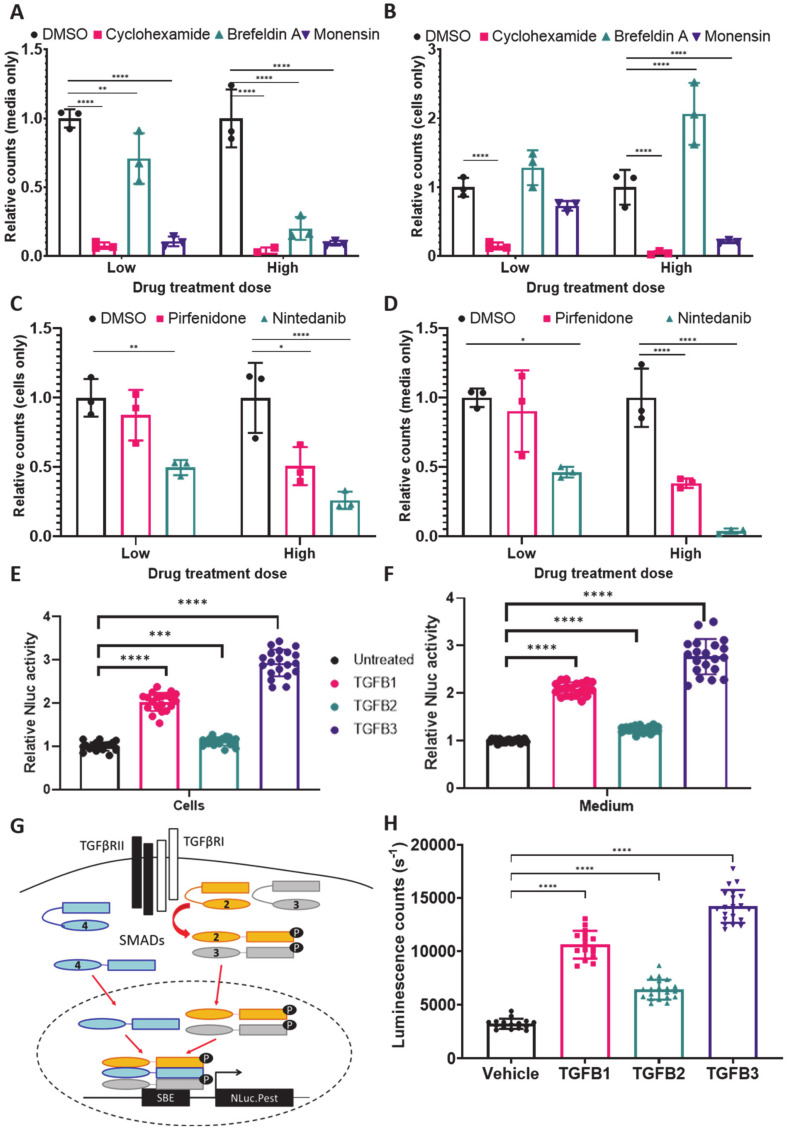
NLuc-PC-I response to known collagen modulators. (**A**) Cellular luminescence in *nluc::Col1a2* cells treated with cycloheximide or the secretion inhibitors Brefeldin A and Monensin after 24 h; the doses used are shown in Table S3. Bars show the mean ± SD for *n* = 3 independent replicate measurements. (**B**) The levels of secreted NLuc after 24 h treatment are shown, demonstrating each treatment results in inhibition of NLuc-PC-I secretion. Bars show the mean ± SD for *n* = 3 independent replicate measurements. (**C**) The effects of the FDA approved therapeutics Nintedanib and Pirfenidone on cellular NLuc activity after 24 h treatment. Bars show the mean ± SD for *n* = 3 independent replicate measurements. (**D**) The effects of Nintedanib and Pirfenidone on NLuc-PC-I secretion with 24 h treatment. Bars show the mean ± SD for *n* = 3 independent replicate measurements. For charts A-D, * denotes *p* < 0.05, ** denotes *p* < 0.01, *** denotes *p* < 0.001 and **** denotes *p* < 0.0001 paired Student’s t-Test. (**E**) Effect of 72 h treatment of *nluc::Col1a2* cells with TGF-β 1, 2, and 3 treatments on cellular NLuc activity. *n* = 5 independent experiments each with four technical repeats. **** indicates *p* = 0.0001 Students paired t-Test, *** indicates *p* = 0.0005 Students paired t-Test. (**F**) The effects of TGF-β on secreted NLuc activity. (**G**) Schematic of SMAD binding element reporter, SMAD2/3 phosphorylation and activation of SMAD4 following binding of TGF-β ligands to the receptor results in recruitment of SMADs to the SBE which drives NLuc-PEST expression. Following flow sorting of stable lentivirus infected cell lines was detected by flow cytometry, Figure S7. (**H**) NIH3T3 stably expressing the SMAD binding element driven NLuc reporter, SBE-NLuc-Pest-RFP, demonstrating robust activation of SMADs after 1 h treatment with TGF-β1 and TGF-β3, a smaller but significant induction of the SMAD reporter was observed with TGF-β2 treatment. *n* = 5 independent experiments each with four technical repeats. **** denotes *p* = 0.0001, paired Student’s t-Test.

Next, we assessed the ability of *nluc::Col1a2* cells to respond to known modulators of collagen-I. As a first experiment, we showed that blocking protein synthesis with cycloheximide brought about a 90% reduction in levels of NLuc-PC-I in conditioned medium and cells (Figure 5A). Likewise, the secretory pathway inhibitors Brefeldin A and Monensin also caused inhibition of NLuc-PC-I secretion (Figure 5A). Treatment with Brefeldin A, unlike Monensin, resulted in accumulation of intracellular NLuc-PC-I (Figure 5B), which is in line with the fact that Brefeldin A is known to induce a strong ER stress response [24]. Encouraged by these results, we next sought to determine if collagen-DyProQ could be used to evaluate the function of the known antifibrotic therapeutics Nintedanib [25] and Pirfenidone [26]. Using doses which did not significantly impact on cell growth (Figure S7A), we observed a reduction in both secreted and cellular NLuc-PC-I (Figure 5C,D). As a further means of evaluating collagen-DyProQ, the *nluc::Col1a2* cells were treated with the profibrotic growth factors TGF-β 1, 2, and 3. Treatment with TGF-β 1 or 3 for 72 h showed strong induction in both cellular and secreted NLuc-PC-1 (Figure 5E,F) without significant effect on cell survival (Figure S7). We transfected NIH3T3 cells with a vector expressing NLuc under the control of a Smad-responsive element (Figure 5G) and flow sorted the transfected cells (Figure S7C,D). The selected cells were then treated separately with TGF-β1, 2, and 3 (Figure 5H). We showed that TGF-β2 had a smaller effect on collagen levels compared to TGF-β1 and TGF-β3, which correlated with the degree of SMAD activation by TGF-β ligands.

## 4. Discussion

In our study, we developed a method for dynamic protein quantitation (DyProQ) of endogenous proteins. CRISPR-Cas9-mediated insertion of NLuc into the target protein of interest is central to the method. Inserting *nluc* into the gene locus ensures that the normal regulatory elements are maintained. Furthermore, the brightness of NLuc in the presence of Furimazine meant that the use of exogenous expression is not necessary. Therefore, DyProQ will be widely applicable to the study of endogenous protein dynamics. Others have measured protein dynamics using fluorescence correlation spectroscopy [27], or by using surrogate markers of transcription [28]; however, these lack scalability, and often require exogenous expression of reporters. Using PC-I as a test protein, we could, with high precision, determine the number of molecules being synthesised, transported in vesicles, and secreted into the culture medium. Collagen-DyProQ is ~10^5^–10^6^ times more sensitive than the conventional chemical method of estimating collagen, and has utility across different platforms, from bioluminescence microscopy to plate reader-style detection for high-throughput screening. Using this method, we quantified PC-I levels in individual cells and up to 20,000 cells, and demonstrated the circadian regulation of PC-I synthesis in fibroblasts and the induction of PC-I in the presence of TGF-β, especially TGF-β3. When imaging the concentration of PC-I in individual cells, we discovered that cells concentrate PC-I in compartments either for storage, degradation, or in preparation for fibril formation.

The site of insertion of NLuc into the target protein sequence is likely to have a bearing on the normal synthesis, trafficking, and secretion of that protein of interest. In our study, we chose to place NLuc at the N-terminus of the proα2(I) chain. The trimeric PC-I molecule comprises two proα1(I) and one proα2(I) polypeptide chains; therefore, each NLuc-PC-I molecule carries one NLuc tag. The assembly and subsequent zippering of the trimeric procollagen molecule is initiated by sequences in the C-terminal of each chain [29]. Furthermore, the major triple helical domain of the molecule is particularly sensitive to mutations that change the repeating Gly-X-Y structure, as shown by studies of osteogenesis imperfecta [30]. Therefore, we chose to insert NLuc at the N-terminus of the molecule, and specifically, in the proα2(I) chain. Green fluorescent protein has previously been located at this position without interfering with trafficking of the protein and subsequent assembly into fibrils [31]. PC-I is converted to collagen by the removal of N- and C-terminal propeptides by procollagen N- and C-proteinases, respectively. Removal of the C-propeptides is required for fibril assembly [32]. However, removal of the N-propeptides is not required for fibril formation [33] and a proportion of collagen molecules retain N-propeptides in the extracellular matrix [34]. Of particular note, failure to remove the N-propeptides of PC-I results in skin hyperextensibility and joint hypermobility in people with the Ehlers-Danlos syndrome type VII [35]. Therefore, in our study, we chose not to engineer out the N-proteinase cleavage site in the proα2(I) chain so as to maintain the physiological functions of the N-propeptide and to approximate, as accurately as possible, the normal synthesis, secretion, and procollagen-handling behaviour of cells. This retention of the endogenous functioning of the procollagen pathway is a key advantage to the DyProQ method, allowing for faithful observations of the normal processes, as well as subsequent perturbations.

The insertion of the GFP11 peptide, a 6 histidine spacer and the NLuc sequences into the N-propeptide of proα2(I) chain, was tolerated by PC-I, as shown by (i) the presence of PC-I in the culture medium, (ii) comparison of PC-I secretion from *nluc::Col1a2* and nonedited cells, and (iii) comparison with published values of PC-I secretion (~200,000 procollagen molecules per cell per hour [36]). The high photon output of NLuc combined with bioluminescence microscopy made it possible to count the number of PC-I molecules in transport vesicles in the secretory pathway, and to record the movement of the vesicles by time-lapse by microscopy. We identified vesicles in which the numbers of NLuc-PC-I molecules remained constant for 20 min, and others in which numbers increased and decreased. These findings provide insights into the possibility that PC-I molecules are delivered to these transport vesicles either for secretion, storage, or degradation. This approach also showed that the concentration of PC-I in some transport carriers was three orders of magnitude higher than the critical concentration of collagen required for fibril formation [32], and five times higher than the surrounding ER concentration. Therefore, cells concentrate procollagen molecules in preparation for collagen fibril formation. Our ability to measure the number of PC-I molecules in individual cells enabled a time-series study of procollagen synthesis, in which we showed that the synthesis of PC-I was rhythmic with a ~24 h period, and thereby confirmed previous proteomic data that the synthesis of PC-I is under circadian clock control [6].

Collagen-DyProQ has immediate applications in studying the synthesis, trafficking, secretion, and degradation of collagen-I caused by mutations in Col1a1 and Col1a2, such as osteogenesis imperfecta, the Ehlers-Danlos syndromes, and Caffey disease. It also has uses in studying the effects on collagen-I synthesis of mutations in genes associated with collagen synthesis, such as FKBP10 and PLOD2 (Bruck syndrome), and BMP1, CREB3L1, CRTAP, P3H1, PPIB, Serpinh1, and TMEM38B (osteogenesis imperfecta). DyProQ could also be used to study the biosynthesis of other collagens, e.g., collagen-II and collagen-XI in Stickler syndrome, collagen-III and collagen-V in the Ehlers Danlos syndrome, collagen-VI in Ullrich congenital muscular dystrophy and Bethlem myopathy, collagen-VII in epidermolysis bullosa, and collagen-IV in sporadic cerebral small vessel disease [37] and major common diseases including stroke (reviewed by [38]). DyProQ has wide-ranging applications in studies of other proteins that are expressed at levels which are too low to be detected by fluorescent protein tagging of the endogenous protein. Finally, mouse models of DyProQ offer the opportunity for whole animal studies.

## Supplementary Materials

The following are available online at https://www.mdpi.com/2073-4409/9/9/2070/s1, Figure S1: RNA validation of successful *nluc* insertion and assessment of cell viability and growth rate, Table S1: Primers used, Video S1: title, Figure S2: Protein validation of successful *nluc* insertion into *Col1* heterotrimer, Table S2: Sequences, Figure S3: Heatmaps of 96-well plates seeded with different numbers of *nluc::Col1a2* cells, Table S3: Drug dosage regimes, Figure S4: Validation of single cell *nluc::Col1a2* NIH3T3 clones, Figure S5: Comparison of luminescence levels from lysed v non-lysed *nluc::Col1a2* cells, Figure S6: rNLuc concentration to luminescence correlation calculations, Figure S7: Cell viability assessment after drug treatment.
